# Relations Between BMI Trajectories and Habitual Physical Activity Measured by a Smartwatch in the Electronic Cohort of the Framingham Heart Study: Cohort Study

**DOI:** 10.2196/32348

**Published:** 2022-04-27

**Authors:** Michael M Hammond, Yuankai Zhang, Chathurangi H. Pathiravasan, Honghuang Lin, Mayank Sardana, Ludovic Trinquart, Emelia J Benjamin, Belinda Borrelli, Emily S Manders, Kelsey Fusco, Jelena Kornej, Nicole L Spartano, Vik Kheterpal, Christopher Nowak, David D McManus, Chunyu Liu, Joanne M Murabito

**Affiliations:** 1 Boston University's and National Heart, Lung, and Blood Institute's Framingham Heart Study Framingham, MA United States; 2 Department of Biostatistics, Boston University School of Public Health Boston, MA United States; 3 Division of Clinical Informatics, Department of Medicine, University of Massachusetts Medical School Worcester, MA United States; 4 Cardiology Division, Department of Medicine, University of California San Francisco San Francisco, CA United States; 5 Section of Cardiovascular Medicine Department of Medicine Boston Medical Center and Boston University School of Medicine Boston, MA United States; 6 Department of Epidemiology Boston University School of Public Health Boston, MA United States; 7 Department of Health Policy & Health Services Research Henry M Goldman School of Dental Medicine Boston University Boston, MA United States; 8 Section of Endocrinology, Diabetes, Nutrition, and Weight Management, Boston University School of Medicine Boston, MA United States; 9 Care Evolution Ann Arbor, MI United States; 10 Cardiology Division, Department of Medicine, University of Massachusetts Medical School Worcester, MA United States; 11 Department of Quantitative Health Sciences, University of Massachusetts Medical School Worcester, MA United States; 12 Section of General Internal Medicine, Department of Medicine Boston University School of Medicine Boston, MA United States

**Keywords:** mobile health, BMI, smartwatch, physical activity, cardiovascular diseases, cardiology, digital health, mHealth, mobile health apps

## Abstract

**Background:**

The prevalence of obesity is rising. Most previous studies that examined the relations between BMI and physical activity (PA) measured BMI at a single timepoint. The association between BMI trajectories and habitual PA remains unclear.

**Objective:**

This study assesses the relations between BMI trajectories and habitual step-based PA among participants enrolled in the electronic cohort of the Framingham Heart Study (eFHS).

**Methods:**

We used a semiparametric group-based modeling to identify BMI trajectories from eFHS participants who attended research examinations at the Framingham Research Center over 14 years. Daily steps were recorded from the smartwatch provided at examination 3. We excluded participants with <30 days or <5 hours of smartwatch wear data. We used generalized linear models to examine the association between BMI trajectories and daily step counts.

**Results:**

We identified 3 trajectory groups for the 837 eFHS participants (mean age 53 years; 57.8% [484/837] female). Group 1 included 292 participants whose BMI was stable (slope 0.005; *P*=.75), group 2 included 468 participants whose BMI increased slightly (slope 0.123; *P*<.001), and group 3 included 77 participants whose BMI increased greatly (slope 0.318; *P*<.001). The median follow-up period for step count was 516 days. Adjusting for age, sex, wear time, and cohort, participants in groups 2 and 3 took 422 (95% CI –823 to –21) and 1437 (95% CI –2084 to –790) fewer average daily steps, compared with participants in group 1. After adjusting for metabolic and social risk factors, group 2 took 382 (95% CI –773 to 10) and group 3 took 1120 (95% CI –1766 to –475) fewer steps, compared with group 1.

**Conclusions:**

In this community-based eFHS, participants whose BMI trajectory increased greatly over time took significantly fewer steps, compared with participants with stable BMI trajectories. Our findings suggest that greater weight gain may correlate with lower levels of step-based physical activity.

## Introduction

The global prevalence of obesity is rising [1], with an estimated 573 million adults projected to be obese by the year 2030 [2]. Obesity is a significant public health problem, and increases the risk of cardiovascular disease (CVD), type 2 diabetes, cancer, and mortality [1,3]. Because obesity at younger ages is associated with negative health outcomes that persist into adulthood [4,5], early intervention may be useful in curbing the adverse outcomes associated with obesity. Lifestyle interventions such as dietary modification and increasing physical activity (PA) levels are used in the management of obesity [6]. Most previous studies that examined the relations between BMI and PA measured BMI at a single timepoint [7-11], ignoring the time-varying nature of BMI. In recent times, the use of trajectories has enabled researchers to track the trends of variables over periods [12,13]. A few studies have reported that BMI trajectories are associated with risk of CVD [14,15]; however, the association between BMI trajectories and PA is less well studied.

The relations between BMI and PA are complex. While some studies suggested an inverse relationship between BMI and PA [7,8,10,16], a few pedometer-based studies produced inconsistent results [7,17,18]. For example, in a study by Tudor-Locke et al [8] to determine the association between ambulatory activity and body composition, higher BMI was correlated with lower daily steps. However, another study by Walker et al [17] did not find any significant association between BMI and PA. Additionally, other studies assessing this relationship were interventional [16,17,19-21], and therefore, the findings may not represent habitual daily walking. Similarly, as most previous studies recruited fewer participants and had short follow-up duration [7,10], the results from these studies may not be generalizable to larger populations and longer follow-up periods. Furthermore, some step-based studies recommended 10,000 steps per day as a PA-promoting measure, and denoted step counts of 5000 or less per day as “sedentary lifestyle index” [22]. The relationship between long-term BMI and habitual step-based PA in community settings remains unclear.

It is thus important to examine the association between BMI trajectories and habitual step-based PA level. Advances in technology permit the use of smaller, light-weight, relatively accessible accelerometers, and allow accelerometer usage in large epidemiological studies [23,24]. As such, the aim of this study was twofold. First, we sought to identify BMI trajectory patterns over 14 years of middle-aged participants enrolled in the electronic cohort of the Framingham Heart Study (eFHS). Second, we aimed to determine the relations between BMI trajectories and daily step count retrieved from a smartwatch.

## Methods

### Study Sample

The details of the Framingham Heart Study (FHS) and the eFHS have been described previously [25,26]. In brief, the FHS enrolled participants in the Third Generation Cohort (Gen 3; n=4095), the multiethnic Omni Group 2 Cohort (n=410), and the New Offspring Spouse Cohort (n=103) from 2002 to 2005. These participants have attended examinations at the research center every 6-8 years. At the time of research examination 3 beginning in June 2016, participants were invited to participate in eFHS if they met the following eligibility criteria: spoke English, had a smartphone, lived in the United States, and were willing to permit notifications and share information with the FHS research center.

Participants who consented to the eFHS study were offered a study smartwatch beginning in November 2016 (Apple Watch Series 0). Of the 3521 participants who attended examination 3, we excluded 1370 who did not provide informed consent for eFHS, including those who had an incompatible phone, and those who had less than 12-month follow-up (n=203). Of the remaining 1948 participants who were enrolled in the eFHS, 1185 chose to participate with the Apple Watch and returned step data between November 2016 and January 2019. We excluded 213 participants who either wore the smartwatch less than 5 hours on any given day or those who returned smartwatch data for less than 30 days during the study period because these participants did not meet the definition for habitual PA previously published [27]. We also excluded 135 participants who did not attend the 3 examinations needed to build the BMI trajectory, participants with BMI values <18.5 or >60 kg/m^2^ at any of the 3 research examinations, and participants who had a gastric bypass procedure. Eligibility and exclusion criteria are depicted in Multimedia Appendix 1.

### Ethics Approval

The study protocol was approved by the Boston University Medical Center Institutional Review Board (H-36586 and H-32132). All participants provided informed consent.

### Body Mass Index Trajectories

At each examination visit in the FHS research center, trained personnel routinely measured participant’s weight to the nearest pound and height to the nearest quarter-inch, using uniform measuring devices. BMI was calculated by dividing the participant’s weight in kilograms by the square of the height in meters (kg/m^2^). Normal weight was defined as BMI within the range of 18.5-24.9 kg/m^2^, overweight as BMI between 25 and 29.9 kg/m^2^, and obese as BMI ≥30 kg/m^2^. To build the BMI trajectories, we included participants in eFHS with Apple Watch data who attended examination 1 (2002-2005), examination 2 (2008-2011), and examination 3 (2016-2019) [25,26]. The median age was 39 (IQR 33-45), 45 (IQR 40-51), and 53 (IQR 47-59) years at examinations 1, 2, and 3, respectively. The median follow-up time for the participants used in BMI trajectories was 14 years (IQR 13-14). We applied a semiparametric, group-based modeling strategy to identify latent homogeneity in BMI trajectories in eFHS participants during their middle adult life. The model assumes the study cohort consisted of a mixture of groups following homogenous developmental courses based on their BMI values [28]. Each participant’s BMI values were centered using his/her baseline measurement to assess change in BMI from examination 1 to 3. The centered longitudinal BMI values were modeled as a mixture of several latent trajectories in a censored normal model (allowing for the lower [–19] and upper [20] BMI limits after centering) with a quadratic function of age. The trajectory models were adjusted for age, sex, and smoking status. We used the SAS “proc traj” program to develop the BMI trajectories. The preferred order of the polynomial (ie, linear or quadratic) for each trajectory and the number of trajectory groups were determined by the Bayesian information criterion (BIC) and the log Bayes factor [28-30]. To identify the optimal number of trajectory groups, we started with a single group, and added 1 more group one at a time. The BIC statistic was used to evaluate the model fit when adding groups.

### Smartwatch Step Data

Participants who consented to the eFHS study using an iPhone were offered Apple Watches Series 0 starting in November 2016 through the end of enrollment. The study research technician assisted the participant with pairing the Apple Watch with her/his iPhone while in the Research Center or provided written instructions for participants who opted to set up the Apple Watch remotely. Participants who attended the research center prior to November 2019 were contacted and provided with the option to return to the Research Center for smartwatch setup or provided with materials for remote setup. Additionally, participants who owned an Apple Watch were permitted to participate using their own watch. The Apple watch has a built-in accelerometer that measures daily steps. All participants were instructed to wear the watch daily. We used daily step counts retrieved from the Apple Watch to assess PA.

### Covariates Obtained at Examination 3

Age, sex, and race/ethnicity were ascertained at examination 1. Educational level and marital status were obtained from self-reports at examination 3. Participants who reported smoking in the year prior to the examination were defined as currently smoking [31]. Prevalent coronary heart disease, myocardial infarction, angina pectoris, stroke, intermittent claudication, and heart failure were classified as CVD, after adjudication by a panel of senior investigators using standard criteria and all available information including hospital records. We defined hypertension as the average of 2 resting blood pressure measurements of ≥140/90 mmHg or a report of antihypertensive medication usage [32]. Type 2 diabetes was defined as fasting glucose ≥126 mg/dL or a report of hypoglycemic agent usage [33]. Sleep apnea was determined based on the self-report of a diagnosis of sleep apnea by a health care professional from technician-administered respiratory questionnaire or the clinical diagnostic impression of the presence of sleep apnea from the standard medical history interview conducted by the nurse practitioner.

### Statistical Analysis

Baseline characteristics of participants were reported as means and SDs for continuous variables and frequencies (percentages) for categorical variables stratified by BMI trajectory groups. We used analysis of variance to compare means of continuous variables and chi-square/Fisher exact test to examine differences in proportions between BMI trajectory groups. The BMI trajectories were created prior to steps assessment. The primary outcome was mean daily steps retrieved from the smartwatch, and BMI trajectories were the primary exposure of interest. We assessed the association between BMI trajectory groups and repeated measures of daily step counts, with BMI trajectory group 1 as the reference group. The statistical analysis was conducted with a generalized linear model that accounted for correlation between longitudinal daily step counts (PROC GENMOD in SAS). We also adjusted for potential confounders. In model 1, the covariates included age, sex, cohort, and wear time. In model 2, we adjusted for hypertensive status, diabetes status, smoking status, and prevalent CVD, in addition to covariates in model 1. In model 3, we adjusted for model 2 plus sleep apnea, education, and marital status. In sensitivity analyses to determine the effect of follow-up duration on daily step count, all models were additionally adjusted for follow-up duration. We used generalized estimating equations to investigate the association between BMI at examination 3 and daily mean steps, adjusting for the same covariates in models 1, 2, and 3.

To investigate whether uneven days of follow-up in the 3 trajectory groups may introduce bias in association analyses, we performed sensitivity analyses by investigating the average of unadjusted daily steps for the participants between the 3 BMI trajectory groups when restricting to a 90-day follow-up period. We performed association analyses between BMI trajectories and mean daily steps within the 90-day periods with model 2. All statistical analyses were performed using SAS version 9.4 (SAS Institute). We defined a 2-tailed *P*<.05 as statistically significant.

## Results

**Figure 1:**
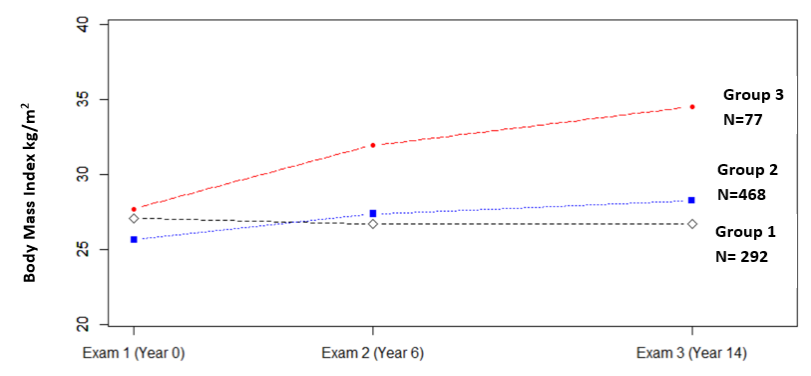
The three trajectory groups based on BMI measures at three health exams for 837 participants in eFHS. eFHS: electronic cohort of the Framingham Heart Study.

The analyses included 837 participants (mean age 53 years; 57.8% [484/837] female) with a median follow-up of 516 days with the maximum follow-up of 1166 days in the eFHS after research examination 3 (ie, the baseline). Based on the comparison of the BIC values and the log Bayes factors from semiparametric group–based models with BMI values from research examinations 1 to 3, the 837 participants were grouped into 3 trajectory groups: group 1 consisted of 292 participants whose BMI remained unchanged (slope [change in BMI for each year] 0.005; standard error [SE] 0.017; *P*=.75) from examination 1 to 3; group 2 included the largest number of participants (n=468) whose BMI slightly increased (slope 0.123; SE 0.014; *P*<.001); and group 3, the smallest group, only included 77 participants who displayed the greatest change in their BMI (slope 0.318; SE 0.029; *P*<.001) from examination 1 to 3 (Table 1 and Figure 1).

**Table 1:** Baseline characteristics of 837 eFHS^a^ participants, by BMI trajectory groups, at examination 3.

Variable		BMI trajectory groups^b^				
		Group 1 (n=292)	Group 2 (n=468)	Group 3 (n=77)	*P*-value^c^	
Age (years), mean (SD)		54 (9)	53 (8)	50 (10)	<.001	
Female, n (%)		166 (56.8)	264 (56.4)	54 (70.1)	.07	
**Race, n (%)**					.44	
	European ancestry	257 (88.0)	425 (90.8)	70 (90.9)		
	Other ancestries	35 (12.0)	43 (9.2)	7 (9.1)		
Hypertension, n (%)		67 (22.9)	116 (24.8)	37 (48.1)	<.001	
Diabetes, n (%)		16 (5.5)	22 (4.7)	6 (7.8)	.52	
Current smoking, n (%)		11 (3.8)	20 (4.3)	5 (6.5)	.58	
Cardiovascular disease, n (%)		14 (4.8)	16 (3.4)	2 (2.6)	.59	
**Self-reported sleep apnea,** **n (%)**					.01	
	Yes	29 (9.9)	55 (11.8)	18 (23.4)		
	No	261 (89.4)	402 (85.9)	59 (76.6)		
**Education, n (%)**					.34	
	Bachelor’s degree or higher	197 (67.5)	327 (69.9)	47 (61.0)		
	No college degree	94 (32.2)	140 (29.9)	29 (37.7)		
**Marital status, n (%)**					.53	
	Married	223 (76.4)	356 (76.1)	54 (70.1)		
	Currently not married	66 (22.6)	110 (23.5)	22 (28.6)		
	Physical Activity Index, mean (SD)	33.6 (4.4)	33.3 (4.9)	33.0 (5.4)	.51	
**BMI, n (%)**					<.001	
	Normal weight	128 (43.8)	115 (24.6)	1 (1.3)		
	Overweight	103 (35.3)	218 (46.6)	15 (19.5)		
	Obese	61 (20.9)	135 (28.8)	61 (79.2)		

^a^eFHS: electronic cohort of the Framingham Heart Study.

^b^Group 1: Participants whose BMI remained stable over the study period; group 2: slight increase in BMI over the study period; group 3: large increase in BMI over the study period.

^c^*P*-value of chi-square test for categorical variables, and detecting if any of the groups are statistically different for continuous variables.

Of the 3 BMI trajectory groups, participants in group 3, on average, were the youngest (mean age 50 years), and contained the largest proportion of women (54/77, 70%). About 48% (37/77) of participants in group 3 had hypertension at baseline (examination 3). In addition, group 3 participants were more likely to have sleep apnea (Table 1). The median follow-up for participants in BMI trajectory group 1 was 576 days (IQR 322-843), for trajectory group 2 was 492 days (IQR 275-790), and for trajectory group 3 was 429 days (IQR 213-717).

A total of 13 participants had missing covariate data for self-reported sleep apnea, 3 had missing covariate data for education, and 6 had missing covariate data for marital status.

We compared the mean daily steps between BMI trajectory groups adjusting for covariates, using the complete case analysis approach (N=815; Table 2). Participants in BMI trajectory group 2 walked 422 fewer steps per day compared with participants in BMI trajectory group 1 (95% CI –823 to –21; *P*=.04), adjusting for age, sex, wear time, and cohort. Participants in BMI trajectory group 3 walked, on average, 1437 fewer steps per day compared with participants in BMI trajectory group 1 (95% CI –2084 to –790; *P*<.001). The effect sizes were slightly attenuated but remained significant after adjusting for cardiovascular risk factors and CVD (model 2; *P*=.04 and <.001 for groups 2 and 3, respectively). The effect sizes were further attenuated after additional adjustment for sleep apnea, education, and marital status (model 3): that is, compared with the reference, groups 2 and 3 walked 382 (95% CI –773 to 10; *P*=.06) and 1120 (95% CI –1766 to –475; *P*<.001) fewer steps per day, respectively (Table 2). The results did not substantially change after additional adjustment for follow-up duration (Multimedia Appendix 2).

**Table 2:** Association between BMI trajectory groups and average daily step count^a^.

Models and groups			Estimate		95% CI		*P*-value		
**Model 1^b^**									
	Group 1^c^ (n=285)	Referent		—		—			
	Group 2 (n=455)	–422		–823 to –21		.04			
	Group 3 (n=75)	–1437		–2084 to –790		<.001			
**Model 2^d^**									
	Group 1	Referent		—		—			
	Group 2	–406		–800 to –12		.04			
	Group 3	–1258		–1908 to –609		<.001			
**Model 3^e^**									
	Group 1	Referent		—		—			
	Group 2	–382		–773 to 10		.06			
	Group 3	–1120		–1766 to –475		.001			

^a^Complete case analysis: n=815.

^b^Model 1 covariates: age, sex, wear time, and cohort.

^c^Group 1: participants whose BMI remained stable over the study period; group 2: slight increase in BMI over the study period; group 3: large increase in BMI over the study period.

^d^Model 2 covariates: model 1 + hypertension, type 2 diabetes, current smoking, and cardiovascular disease.

^e^Model 3 covariates: model 2 + sleep apnea, education, and marital status

We assessed the association between BMI at examination 3 and mean daily steps (Multimedia Appendix 3). Higher BMI values were associated with lower mean daily step counts. For every kg/m^2^ increase in BMI at examination 3, mean daily step count decreased by 146 (95% CI –182 to –111; *P*<.001) steps, after adjusting for age, sex, wear time, cohort, hypertension, diabetes, current smoking, CVD, sleep apnea, education, and marital status.

**Figure 2:**
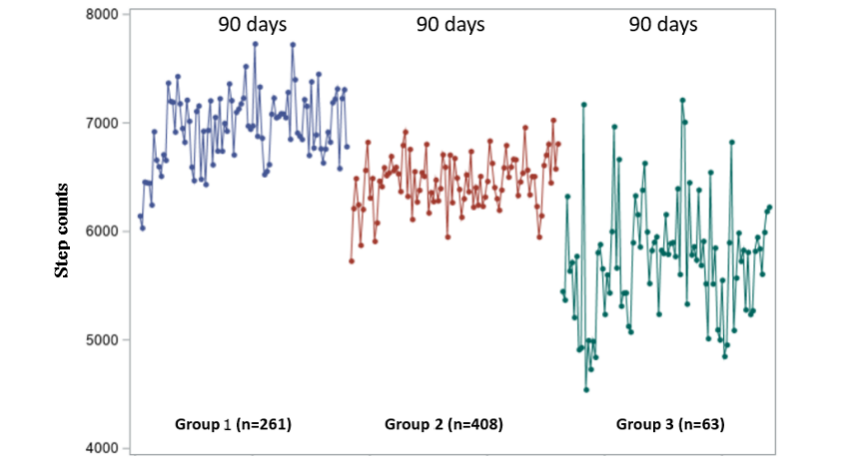
Median daily step distributions of BMI trajectory groups at 90-day window.

In sensitivity analyses, we investigated the daily median steps among the participants in BMI trajectory group 1, group 2, and group 3 during the 90-day follow-up without adjusting for covariates (Figure 2). BMI trajectory group 1 displayed the highest median step value within the 90-day period (6898 steps; IQR 4242-10298) and BMI trajectory group 3 participants had the least median daily steps (5707 steps; IQR 3335-8668; Figure 2). After adjusting for covariates, the differences in PA by BMI trajectory group remained similar during the 90-day follow-up period compared with the 12-month period. Within the 90-day interval, participants in BMI trajectory group 2 walked, on average, 659 fewer steps per day compared with those in BMI trajectory group 1 (95% CI –1124 to –194; *P*=.01), adjusting for age, sex, wear time, cohort, and cardiovascular risk factors. Similarly, participants in BMI trajectory group 3 walked, on average, 1006 fewer steps per day compared with participants in BMI trajectory group 1 (95% CI –1847 to –286; *P=*.01; Multimedia Appendix 4).

## Discussion

### Principal Findings

In this community-based electronic cohort of middle-aged and older participants, we examined the relations between BMI trajectories that were constructed at midlife and daily steps captured from a smartwatch worn over 1 year. We identified 3 distinct trajectory groups for BMI change over 14 years. The BMI change trajectory remained relatively stable for 34.9% (292/837) of participants; the majority of participants (468/837, 55.9%) had slight increments in BMI, whereas 9.2% (77/837) of participants had high increments in their BMI. We observed that in adjusted analyses, participants who had slight and greater increments in BMI took fewer steps per day, compared with participants whose BMI remained stable.

Our findings were consistent with previous studies demonstrating that individuals with higher weight took fewer mean daily steps compared with those with lower weight [7,18,34]. For instance, in an accelerometer-based cross-sectional study of 108 adults, participants with obesity took significantly fewer steps compared with those with normal weight [35]. Although the accelerometer usage overcame a major limitation of self-reported PA, BMI was measured from a single timepoint, and follow-up was relatively short. In another study of 1006 adolescents, Nesbit et al [36] demonstrated that participants within higher BMI trajectory groups were less physically active, compared with participants within healthy BMI trajectory groups. Similarly, a study of 3070 middle-aged Canadians showed that being physically active was associated with a lower risk of being in the overweight and obese trajectory groups [37]. In a more recent study, Laranjo et al [38] demonstrated that participants who were underweight/normal weight took significantly more steps per day over a 6-month period while those who were overweight/obese did not show any significant changes [38]. Although this study examined BMI and step count concurrently [38], the sample size may have been too small to detect significant changes among those in the overweight/obese group. In another study that found a negative association between daily step count and BMI, daily steps were measured over a relatively shorter period and BMI was measured only at 1 timepoint [39]. Although higher baseline BMI (at examination 3) was associated with significantly fewer mean daily steps, BMI trajectories factored in several objective measurements of BMI rather than self-reported height and weight in other published reports, or single BMI measurement. In addition, multiple objective measurement of step count, with a follow-up period of 1 year or more, adds more to understanding the relation between BMI and step-based PA. In this study, we show that higher levels of step-based PA are correlated with maintaining a stable BMI over time.

We observed that the smallest proportion of total participants (trajectory group 3) had the greatest (sharpest) increment in BMI over the period. This is consistent with the trend reported in previous trajectory studies [34,37,40,41]. For instance, in a recent study of 3271 young-to-middle-aged adults, 1.9% of participants were in the sharply rising BMI trajectory group, while about 46% of participants maintained a low-to-stable BMI [40].

Lifestyle factors may account for the different trajectories we observed. For example, in a 4-year lifestyle study of 120,887 men and women, Mozaffarian et al [42] found that diet, PA, alcohol, and smoking were associated with long-term weight gain. In our study, a greater proportion of participants in BMI trajectory group 3 were female and younger compared with the other BMI trajectory groups, and had a higher prevalence of hypertension. Furthermore, only 1 participant in BMI trajectory group 3 had normal weight and the rest of the participants were either overweight or obese. This is consistent with findings from some previous studies [34,40,41]. Life events such as pregnancy and motherhood may account for weight gain among young and middle-aged women [43]. Similarly, obesity is associated with hypertension [44], and may explain the high prevalence of hypertension among participants in BMI trajectory group 3. Because BMI trajectories in early adulthood increased risk for incident hypertension [40], early identification of individuals with higher risk of higher BMI trajectories may provide an opportunity to decrease obesity and reduce hypertension risk. It is possible that these lifestyle factors account for the trends we observed among participants who gained the most weight over the study period. Therefore, younger adults, particularly females, could benefit from early lifestyle interventions to prevent excessive weight gain over time.

This study included eFHS participants who had the opportunity to contribute at least one year of follow-up step data; however, due to the rolling enrollment design of the eFHS, some participants had the ability to contribute even longer data, while others may have dropped out during follow-up. As such, the follow-up duration was different among the BMI trajectory groups, with participants in BMI trajectory group 1 recording the longest follow-up duration (median 576 days), whereas those in trajectory group 3 the shortest (median 429 days). While it is possible that this observation may have an effect on the daily step count recorded, effect sizes were only slightly attenuated but remained significant after additional adjustment for follow-up duration in the models.

The strengths of this study include the moderate-size community-based sample of eFHS participants, the standardized and objective assessment of BMI used in building BMI trajectories over mid-adulthood, and step count data gathered with a smartwatch for a median of 1 year, providing abundant step data for analyses. Covariates were well characterized and directly measured at the FHS research center. Furthermore, because we excluded participants with BMI values <18.5 or >60 kg/m^2^, as well as those who had a gastric bypass, we reduced the number of outliers in our analysis.

There are some limitations of our study to consider. First, because our analysis included a majority of European ancestry participants, our findings may not be generalizable to people of different race/ethnicity. Second, because eFHS participants were healthier, well educated, and had higher socioeconomic status compared with the overall FHS examination attenders, it is possible that their level of PA may differ from people of lower educational level and socioeconomic status. Moreover, because we excluded participants who returned data for <30 days, it is possible our findings may differ in this group. Although we adjusted for known confounders, our study may be subject to residual confounding. Because trajectories were created before comparison with step data, it is possible that other factors, besides PA, such as diet or illness, may have contributed to the trajectories we observed. As the study did not measure other types of PA besides step count, it is possible participants in the different BMI trajectory groups performed other types of PA that may affect weight change. Lastly, because the study was observational, causality cannot be inferred.

### Conclusions

In this community-based study of eFHS participants, participants whose BMI trajectory increased greatly prior to step count measurement took significantly fewer daily steps, compared with participants with stable BMI trajectories. Our findings suggest that greater weight gain may be associated with lower levels of step-based PA during adulthood. Future research should investigate the long-term trends of other lifestyle factors such as diet and smoking, and assess the relationship between these factors and habitual PA.

## Appendix

Multimedia Appendix 1 Study sample selection.

Multimedia Appendix 2 Association between BMI trajectory groups and average daily step count, additionally adjusted for follow-up duration.

Multimedia Appendix 3 Association between BMI at exam 3 and average daily step count.

Multimedia Appendix 4 Association between BMI trajectory groups and average daily step count for individuals at the 90-day window.

## Abbreviations

BIC: Bayesian information criterion
CVD: cardiovascular disease
eFHS: electronic cohort of Framingham Heart Study
FHS: Framingham Heart Study
SE: standard error
