# Supplementary material for: Relations Between BMI Trajectories and Habitual Physical Activity Measured by a Smartwatch in the Electronic Cohort of the Framingham Heart Study: Cohort Study
Source: JMIR Cardio. 2022 Apr 27;6(1):e32348. doi: 10.2196/32348 (PMC9096636; doi:10.2196/32348)
Supplement: Multimedia Appendix 1 [file cardio_v6i1e32348_app1.docx]

**Multimedia Appendix 1.** Study sample selection.

Third Generation, the multi-ethnic Omni Group 2 and the New Offspring Spouse cohort participants who attended Exam 3 (April 2016 to April 2019)

N=3521

Participants not eligible for the eFHS study **(n=1573)**

Had less than 12 months follow-up (n=203)

Did not consent or had incompatible phone (n=1370)

Participants who participated in eFHS cohort

N=1948

Participants ineligible for the Apple Watch step study **(n=763)**

- Did not take the Apple Watch (n=710)
- Never returned data (n=53)

Participants who returned Apple watch step data

N=1185

BMI and step exclusions **(348):**

Wearing smart watch <5 hours in a day or wearing for <30 days (N=213)

Failed to attend three health exams or BMI values < 18.5 or > 60 kg/m^2^ (N=135)

Final study sample

N= 837
