# Supplementary material for: Relations Between BMI Trajectories and Habitual Physical Activity Measured by a Smartwatch in the Electronic Cohort of the Framingham Heart Study: Cohort Study
Source: JMIR Cardio. 2022 Apr 27;6(1):e32348. doi: 10.2196/32348 (PMC9096636; doi:10.2196/32348)
Supplement: Multimedia Appendix 2 [file cardio_v6i1e32348_app2.docx]

**Multimedia Appendix 2. Association between BMI trajectory groups and average daily step count, additionally adjusted for follow-up duration**

| **Model 1^a^** | | | |
| --- | --- | --- | --- |
| **Groups^b^** | **Estimate** | **95 % Confidence Interval** | ***P* value** |
| **Group 1 (N=285)** | referent | ---------- | ---------- |
| **Group 2**  **(N=455)** | -408 | -810, -6 | .05 |
| **Group 3**  **(N=75)** | -1414 | -2061, -766 | <.001 |
| **Model 2^c^** | | | |
| **Group 1** | referent | ---------- | ---------- |
| **Group 2** | -393 | -788, 1 | .05 |
| **Group 3** | -1240 | -1889, -590 | <.001 |
| **Model 3^d^** | | | |
| **Group 1** | referent | ---------- | ---------- |
| **Group 2** | -371 | -763, 21 | .06 |
| **Group 3** | -1107 | -1752, -461 | <.001 |

Complete case analysis: N= 815

^a^ Model 1 covariates: age, sex, wear time, follow-up duration and cohort.

^b^Group 1: Participants whose BMI stayed stable over study period; Group 2: Slight increase in BMI over study period; Group 3: Large increase in BMI over study period

^c^ Model 2 covariates: model 1 + hypertension, type 2 diabetes, current smoking, and cardiovascular disease.

^d^Model 3 covariates: model 2 + sleep apnea, education, and marital status
