# Supplementary material for: Relations Between BMI Trajectories and Habitual Physical Activity Measured by a Smartwatch in the Electronic Cohort of the Framingham Heart Study: Cohort Study
Source: JMIR Cardio. 2022 Apr 27;6(1):e32348. doi: 10.2196/32348 (PMC9096636; doi:10.2196/32348)
Supplement: Multimedia Appendix 3 [file cardio_v6i1e32348_app3.docx]

**Multimedia Appendix 3. Association between BMI at exam 3 and average daily step count**

| **Model 1^a^** | | | | |
| --- | --- | --- | --- | --- |
|  | **Estimate** | **Standard Error** | **95 % Confidence Interval** | ***P value*** |
| **BMI at exam 3** | -158 | 17 | -191, -124 | <.001 |
| **Model 2^b^** | | | | |
| **BMI at exam 3** | -149 | 18 | -184, -114 | <.001 |
| **Model 3^c^** | | | | |
| **BMI at exam 3** | -146 | 18 | -182, -111 | <.001 |

Complete case analysis: N =815

^a^ Model 1 covariates: age, sex, wear time and cohort.

^b^ Model 2 covariates: model 1 + hypertension, type 2 diabetes, current smoking, and cardiovascular disease.

^c^Model 3 covariates: model 2 + sleep apnea, education and marital status
