# Supplementary material for: Relations Between BMI Trajectories and Habitual Physical Activity Measured by a Smartwatch in the Electronic Cohort of the Framingham Heart Study: Cohort Study
Source: JMIR Cardio. 2022 Apr 27;6(1):e32348. doi: 10.2196/32348 (PMC9096636; doi:10.2196/32348)
Supplement: Multimedia Appendix 4 [file cardio_v6i1e32348_app4.docx]

**Multimedia Appendix 4. Association between BMI trajectory groups and average daily step count for individuals at the 90-day window**

| **Model^a^** | | | |
| --- | --- | --- | --- |
| **Group 1** | referent | ---------- | ---------- |
| **Group 2** | -659 | -1124, -194 | 0.006 |
| **Group 3** | -1066 | -1847, -286 | 0.007 |

N = 732

Group 1: Participants whose BMI stayed stable over study period; Group 2: Slight increase in BMI over study period; Group 3: Large increase in BMI over study period

^a^ Model covariates: age, sex, wear time, cohort, hypertension, type 2 diabetes, current smoking, and cardiovascular disease.
